# Prevalence and Determinants of Peripheral Neuropathy among Type 2 Adult Diabetes Patients Attending Jimma University Medical Center, Southwest Ethiopia, 2019, an Institutional-Based Cross-Sectional Study

**DOI:** 10.1155/2020/9562920

**Published:** 2020-06-29

**Authors:** Daba Abdissa, Nigusse Hamba, Kumsa Kene, Deriba Abera Bedane, Guluma Etana, Dassalegn Muleta, Asfaw Gerbi

**Affiliations:** ^1^Department of Biomedical Sciences (Clinical Anatomy), College of Medical Sciences, Institute of Health Sciences, Jimma University, Ethiopia; ^2^Department of Biomedical Sciences (Medical Biochemistry), College of Medical Sciences, Institute of Health Sciences, Jimma University, Ethiopia; ^3^Department of Biomedical Sciences (Medical Physiology), College of Medical Sciences, Institute of Health Sciences, Jimma University, Ethiopia; ^4^Department of Biomedical Sciences (Clinical Anatomy), College of Medical Sciences, Wollega University, Ethiopia; ^5^Department of Medical laboratory Sciences (Medical Microbiology), College of Health Sciences, Mizan-Tepi University, Ethiopia

## Abstract

**Background:**

Diabetes chronic complications are major causes of morbidity and mortality, among which diabetic peripheral neuropathy (DPN) stands out. One of the tools to screen DPN is the Michigan neuropathy screening instrument. However, there is no data compiled using this tool to assess the prevalence and its determinants in Jimma. So, the aim of this study was to assess the prevalence of DPN and its determinants among patients with diabetes mellitus at Jimma University Medical Center*. Methods*. A hospital-based cross-sectional study was conducted at Jimma University Medical Center on 366 type 2diabetic patients. Data were collected using pretested structured questionnaire and entered into EpiData 3.1 and exported to SPSS version 20 for analysis. Both bivariate and multivariate binary logistic regressions were employed to identify factors associated with DPN. A variable having a *p* value of < 0.25 in the bivariate model was subjected to multivariate analysis to avoid confounding variable's effect. Adjusted odds ratios were calculated at 95% confidence interval and considered significant with a *p* value of ≤ 0.05.

**Results:**

The mean age of participants was 50.1 ± 14.28 years. The study finding showed that the prevalence of DPN was 53.6% among study participants. According to the multivariate logistic regression age above 40 years (AOR = 4.57; 95% CI: 1.50, 13.9), above 50 years (AOR = 6.5; 95% CI: 2.24, 18.79), duration of diabetes above 5 years (AOR = 3.06; 95% CI: 1.63, 5.77), duration above 10 years (AOR = 7.1; 95% CI: 2.99, 17.28), physical inactivity (AOR = 2.02; 95% CI: 1.14, 3.55), and smoking (current smoker AOR = 7.96, 95% CI: 3.22, 19.64; former smoker (AOR = 2.65; 95% CI: 1.22, 5.77) were independent predictors of DPN among study participants.

**Conclusion:**

Almost half of the study participants had DPN. Age above 40 years, diabetes duration of above 5 years, physical inactivity, and smoking were significantly associated with DPN. Early detection and appropriate interventions are important among patients with age above 40 years, physically inactive, smokers, and diabetes duration of above 5 years.

## 1. Introduction

Globally, diabetes mellitus (DM) is now one of the most common noncommunicable diseases and is the leading cause of death in most developed countries [1]. Worldwide, around 387 million people have DM according to the International Diabetes Federation (IDF) update of 2014 [2]. World health organization (WHO) estimates the number of cases of diabetics in Ethiopia to be about 800,000 in 2000 and projected that it would increase to about 1.8 million by the year 2030 [3]. DM is the main underlying cause of blindness, renal failure, peripheral neuropathy with subsequent lower extremity amputation, and even death [4, 5].

Diabetic peripheral neuropathy (DPN) is the most common complication of DM, and it is estimated that 30% to 50% of diabetes patients are affected by this disorder [6, 7]. The Toronto Consensus Panel defined DPN as a symmetrical, length-dependent sensorimotor polyneuropathy attributable to metabolic and microvessel alterations as a result of chronic hyperglycemia exposure [8]. DPN occurs in 60–70% of the 347 million people living with DM worldwide [9]. In Africa, the prevalence is higher due to late diagnosis, scarcity of screening and diagnostic resources, poor control of blood sugar, and other precipitating factors [10, 11].

DPN leads to a number of impairments and functional limitations, including foot ulceration and subsequent lower extremity amputation, fifteen times increase of the falling risk, inability to work due to physical limitations, and frequent hospitalizations [7, 12–15]. It is estimated that every 30 seconds a lower limb or part of a lower limb is lost somewhere in the world as a consequence of diabetes [16, 17]. In the presence of neuropathy the annual incidence of foot ulceration has demonstrated an approximately 10-fold increase [18]. A recent study in Jimma showed that the prevalence of foot ulcer in diabetic patients on follow-up at Jimma Medical Center was 11.6% and peripheral neuropathy is one of the major risk factor identified for diabetic foot ulcer [19].

About 25% of people with type 2 diabetes have evidence of diabetic complications at the time of initial diagnosis [20]. More than 50% of DPN patients are found to be asymptomatic [21], and prevalence of neuropathy is estimated to be about 8% in newly diagnosed patients and greater than 50% in patients with longstanding disease [22]. According to several studies, the most common risk factors associated with DPN includes advanced age, long duration of diabetes, poor glycemic control, and cigarette smoking [23–25].

Screening DM patients for DPN at the earliest stage is important to minimize the damaging effects of its serious complications. This DPN screening for community and outpatient settings also successfully predicts those at risk for diabetic foot ulcer. Moreover, modern equipment is less likely available to care for DPN patients in developing countries [26]. Therefore, early screening of DPN and its determinants is very crucial especially for developing countries with low resource settings and low educational status.

Despite DPN being a significant complication associated with DM, there are no data compiled in Jimma University Medical Center (JUMC) which evaluates screening for DPN in diabetic patients. Therefore, the purpose of this study is to investigate the prevalence and factors associated with DPN among diabetic patients on follow-up at JUMC.

## 2. Methods and Materials

### 2.1. Study Setting, Period, and Design

An institution-based cross-sectional study was conducted on 366 type 2 diabetic patients on follow-up at JUMC, Southwest Ethiopia, from September 1 to November 30, 2019. JUMC is one of the largest referral hospitals in our country serving a very large catchment area in the Southwestern Oromia region as a referral hospital which is located about 352 kilometer to southwest of Addis Ababa.

### 2.2. Population

The source population comprised of all adult type 2 diabetic patients who were under routine follow-up clinic of JUMC, while the study population comprised of type 2 diabetic patients who were under routine follow-up clinic of JUMC who fulfilled the inclusion criteria.

### 2.3. Eligibility Criteria

Participants of age ≥ 18 years were included and those who were critically ill, DM patients with HIV, leprosy, type 1 DM, peripheral nerve injury, unable to hear and see, and bilateral lower extremity amputees were excluded based on medical chart, physical assessment, history, and laboratory test.

### 2.4. Sample Size Calculation and Sampling Technique

The sample size was calculated using a single population proportion formula by considering the prevalence of diabetic peripheral sensory neuropathy in Bahirdar, Ethiopia, as 52.2% [24], at 95% CI and a margin of error 5%. It gives an initial sample size of 383. Since the source populations of diabetic patients at the clinic were less than 10,000, we employed a population correction/adjustment formula for a finite population. Then, the final sample size was calculated to be 333. By adding a 10% nonresponse rate, it gave the final sample size of 366. A systematic random sampling technique was employed to select study participants.

### 2.5. Data Collection Tool and Procedure

Data were collected by using a structured questionnaire through face-to-face interviews, patient record reviews, and physical examination. The questionnaire was adapted from WHO stepwise approach for surveillance of chronic disease risk factors [27] and from different scientific journals. The questionnaire contains sociodemographic factors, Michigan neuropathy screening instrument, behavioral variables, and clinical variables.

Michigan neuropathy screening instrument (MNSI) was used to evaluate the presence of DPN. It is a well-known instrument used to assess peripheral neuropathy among patients with type 2 DM with a sensitivity of 80% and a specificity of 95%. It is a validated, noninvasive, and inexpensive measurement tool that evaluates sensory and motor components of neuropathy which contains history and physical assessment parts [28, 29].

### 2.6. MNSI History Version

It contains 15 items which was administered by the interviewer, of which 13 items assess symptoms of DPN while item #4 is a measure of impaired circulation and item #10 is a measure of general asthenia, hence were not included in scoring. ‘Yes' responses to questions 1–3, 5, 6, 8, 9, 11, 12, 14, and15 were each counted as one point and ‘No' responses to questions 7 and 13 likewise counted as one point. The total score ranges from 0 to 13 points and a score of ≥7 indicated the presence of DPN.

### 2.7. MNSI Examination Version Procedure and Scoring

It assessed the following five variables on each foot which was performed by data collectors:

1. Each foot was inspected for deformities, dry skin, and calluses or infections, and each foot with any abnormality received a score of one and if not 0
2. Each foot was inspected for ulcer, and each foot with an ulcer received a score of 1 and if not 0
3. Examination of vibration sense by tuning fork 128 hertz

A 128-hertz (HZ) C-tuning fork made in USA was used to detect loss of vibration. Vibration sensation was tested in the great toe and the score was designated as follows for each foot. Vibration was present and scored 0 if the examiner senses the vibration on his finger for <10 seconds, 0.5 score when the examiner felt the vibration for ≥10 seconds after the patient stopped to feel it at the great toe, and 1 point for absent vibration.

(4) Detection of the ankle reflex

Ankle reflex function was detected using standard triangular rubber-headed hummer made in China. If ankle reflex was present, it was scored as 0, while if absent the patient was asked to perform the Jendrassic manoeuver and if present it was designated present with reinforcement and scored 0.5. Finally, if absent with the Jendrassic manoeuver, the reflex was designated absent and scored 1.

(5) Monofilament test

A 5.07/10 g monofilament made in China was used to detect loss of pressure sensation on the feet. It is an objective instrument used in screening the diabetic foot for loss of protective sensation. Each monofilament was used to test 10 patients to avoid diagnosing error. The participant, whose eyes were closed, was asked to respond yes if he/she felt the filament. Nine sites on the plantar surface of the foot and one on the dorsum were tested. 8 correct responses out of 10 applications was considered normal and scored 0, 1-7 correct responses indicates reduced sensation and scored 0.5, and no correct answers translated into absent sensation and scored 1.

Both feet were independently assessed and the scores for both feet were added together. After summing up all the components, the patient was considered to have DPN if the score was ≥2.5 out of the 10-point scale on examination version of MNSI.

Weight was measured using a standard weight scale in kg approximated to the nearest 0.1 kg reading by leveling at 0 for each subject. Height was measured using a standard height measurement scale in meters with upright standing position and was approximated to the nearest 0.1 cm reading. Behavioral variables were assessed based on the WHO stepwise approach for chronic disease risk factor surveillance [27]. Body mass index (BMI) was calculated as weight (kg)/height (m^2^). Patient records (charts) were used to take clinicalvariables. Data collection was carried out by three medical interns and one physiotherapist with the supervision of a principal investigator and 2 supervisors.

### 2.8. Operational Definition

#### 2.8.1. Diabetic Peripheral Neuropathy

Diabetic peripheral neuropathy: was present if the patient's history version of MNSI questionnaire score was ≥7 abnormal responses in the legs and/or if the lower extremity examination version of MNSI scores was ≥2.5.

#### 2.8.2. Amputation

Amputation is the surgical removal of the whole or a part of the limb including its distal end.

#### 2.8.3. Critically Ill

Patients who are unable to communicate and abnormal conscious are considered critically ill.

#### 2.8.4. DM Duration

The duration of DM was calculated as age at data collection minus age at onset of DM.

#### 2.8.5. Physically Inactive

Patients performing moderate-intensity activity less than 150 minutes per week were considered physically inactive.

#### 2.8.6. Physically Active

Patients performing moderate intensity more than 150 minutes per week were considered physically active.

### 2.9. Data Entry, Processing, and Analysis

After coding and checking for completeness and consistency, data were entered into EpiData version 3.1 and then were exported to SPSS version 20 for analysis. First, frequency distributions of variables were explored, and descriptive statistics were used to summarize and present the information in the form of mean, median, percentages, and tables with 95% confidence intervals for prevalence estimates. A binary logistic regression model with backward likelihood ratio procedure was used to examine factors associated with DPN among the study participants. Variables which showed association with a dependent variable in the bivariate analyses at *p* < 0.25 were entered into the multivariate logistic regression model. Multiple binary logistic regression analysis was used to examine the association between independent variables and dependent adjusting for other potential confounders and a *p* value of ≤ 0.05 was used to define statistical significance. The Hosmer and Lemeshow goodness-of-fit test were checked and gave a *p* value of 0.63, indicating evidence of fitness of the model. *p* value ≤ 0.05 was taken as statistically significant.

### 2.10. Data Quality Assurance

Data quality was ensured through standardized data collection materials and the English version questionnaire was translated to Amharic version for interviewing and back to English after data collection. The original and translated questionnaires were compared, and the discrepancies were reviewed and resolved accordingly. Pretest on 5% (18 subjects) of the sample population was conducted at Shenen Gibe Hospital Diabetic Clinic, and amendment of the questionnaire was done. After analyzing pretest results, necessary modifications and corrections were made. Two days of training was given for data collectors about the aim of the study, on the ways of data collection, and how to measure, observe, and record readings before data collection was undertaken. Continuous follow-up and supervision was made by the 2 supervisors and principal investigator throughout the data collection period. The collected data were checked on daily basis for accuracy and completeness by principal investigator and supervisors.

To minimize intra- and interexaminer variation on physical examination, at first we have made a detailed training on each step of the physical examination procedure; all the data collectors were well informed to strictly follow the guideline during procedure; continuous supervision was conducted and each physical examination was crosschecked by 2 supervisors independently.

## 3. Result

### 3.1. Sociodemographic Characteristics of Participants

During the study period, a total of 366 participants were included. More than half (55.5%) of the respondents were males. The mean age of the respondents was 50.1 ± 14.28 years. Many of the respondents (44.3%) were Muslims, and almost half of the participants were rural dwellers (51.6%). Regarding the marital status of the respondents, more than three-fourth (79%) was married (Table 1).

### 3.2. Clinical and Behavioral Characteristics of Participants

Among study participants, almost two-third (66.7%) of them was diagnosed with diabetes for ≥10 years. A total of 231 (63.1%) study participants were in the normal category of BMI, whereas 84 (23%) of the participants were overweight. Among the total participants, almost three-fourth (77.6%) used a noninsulin drug and 54 (14.8%) participants were active smokers currently, while 244 (66.7%) had never smoked (Table 2).

### 3.3. Prevalence of Diabetic Peripheral Neuropathy

In this study, the composite MNSI symptom and sign scores were used for defining prevalence of DPN for each study subject. Neuropathy was then defined as score of ≥7 (out of 13) on the MNSI questionnaire for neuropathic symptoms and/or score of ≥2.5 (out of 10) on examination for signs of neuropathy. Accordingly, the overall prevalence of DPN among the study population was 53.6% (seen in 196 out of 366, in almost half of the respondents). Based on the symptom score alone, the prevalence of DPN was 69 (18.9%), while that of the sign score was 187 (51.1%).

### 3.4. Factors Independently Associated with DPN

On bivariate evaluation, six variables showed evidence of some association with the outcome at a *p* value of *<* 0.25, hence included in the multivariate logistic regression analysis. From those variables; 1^st^ age of respondent was one of the independent factors in predicting the DPN. Participants in their 5^th^ decade (40-49 years) were 4.57 times more likely to develop DPN compared to patients younger than 30 years (*AOR* = 4.57; 95% CI: 1.5, 13.9) controlling for all other factors in the model. Furthermore, participants aged 50 years and older were 6.5 times more likely to develop DPN compared to patients whose age was less than 30 years (AOR = 6.5; 95% CI:2.24, 18.79) provided that all other variables remain the same. The other factor identified was diabetic duration. Participants of 5 to 10 year duration of DM were 3.06 times more likely to develop DPN as compared with those who with less than 5 years duration of DM (AOR = 3.06; 95% CI: 1.63, 5.77). Those participants of greater than 10 year duration were 7.19 times more likely to develop DPN as compared with those with shorter diabetic history less than 5 years (AOR = 7.19; 95% CI: 2.99, 17.2) after controlling for other variables.

Similarly, diabetic patients who were currently active smokers were 7.96 times more likely to develop DPN as compared with those who never smoked (AOR = 7.96; 95% CI: 3.22, 19.64). Likewise, former smokers were 2.65 times more likely to develop DPN as compared with those who never smoked (AOR = 2.65; 95% CI: 1.22, 5.77). Finally, diabetic patients who were physically inactive were 2.02 times more likely to develop DPN as compared with physically active respondents (AOR = 2.02; 95% CI: 1.14, 3.55) (Table 3).

## 4. Discussion

The current study intended to determine the prevalence of DPN and its correlates in adult patients with type 2DM. According to our study finding, the prevalence of DPN as per MNSI history version was 18.9%, which was much less than THE prevalence explored by MNSI examination of the same study which turns to be 51.1%. This difference shows the limitations related to patients self-perception of symptoms of DPN and suggests insisting on examination version of MNSI. The overall prevalence of DPN among the study participants based on MNSI was 196 (53.6%) from the total of 366 participants (95% CI 48.9, 59). Our study result was in line with one study from Ethiopia, 52.2% in Bahirdar [24]. Similarly, the prevalence in the current study was also consistent with prevalence reported from other countries like Yemen, the USA, Ghana, and Malaysia where the prevalences were found to be 56.2%, 51%, 50.7%, and 50.7%, respectively [30–33]. The possible explanation for similarity of our study result with the study done in Bahirdar, the USA, and Ghana could be due to the use of the same tool and with the study in Yemen and Malaysia might be due to same study design.

On the other hand, the present study finding was higher than a study conducted in Jordan which was 39.5% [34]. This variability might be due to difference in genetic predisposition and difference in health care qualities. Likewise, a study conducted in China showed that the prevalence of DPN was 33.1% [35]. The observed higher DPN prevalence rate in our study could be the result of different medical care access, genetic predisposition, and population studied. Moreover, studies from Libya 30.5% [36] and from Mulago, Uganda, 29.5% [37] show lower prevalence of DPN. These discrepancies in the prevalence most likely reflect difference in the study population used, difference in diagnostic criteria employed, and different methods of participant selection.

Diabetic peripheral neuropathy prevalence reported from Mekelle and Jimma, Ethiopia, were 41% [38] and 25.4% [39], respectively. The possible reason for the difference in the prevalence of DPN between our study and the one from Mekelle could be due to the difference in the study design, i.e., the study from Mekelle used a prospective cohort study of 6-week duration which detects only new cases. In the previous study reported from Jimma, the information about DPN was obtained from the patient chart review, sample size was small, and both type 1 and 2 DM were included. Because DPN may not be routinely registered on patient chart during follow-up, this could be the cause of missing cases with DPN in the previous study. In our study, DPN was assessed by using MNSI history version and examination version which can identify more cases.

However, the current study showed lower prevalence of DPN when compared to other studies reported from Iran, Nigeria, and Italy with DPN prevalence of 75.1%, 75%, and 82%, respectively [40–42]. The higher prevalence of those studies could be due to difference in study settings, tool used, and study design. For instance, Nigerian study used vibration perception threshold tool of greater than 15 volts which might overestimate the prevalence of DPN, whereas the Iranian study used a large sample size and used nerve conduction velocity which is the gold standard test. Finally, the disparity of the current study from a study conducted in Italy might be due to difference in tools used, study design, and genetic predisposition.

According to the current finding, patient's age above 40 years was an independent predictor of DPN. This report was supported by previous studies [24, 25, 33, 43]. Possible reasons for this association could be justified as peripheral neuropathy is a chronic complication of diabetes and takes time to develop, so it is expected in older diabetic patients. In addition with aging, the nervous system is increasingly vulnerable to continual metabolic stress and degenerative nature of physiological well-being [43].

In our study finding, we found that DM duration of greater than 5 years was found to be significantly associated with DPN. Various study findings also reported the same result [24, 25, 33, 34]. This association can be explained by longer duration of diabetes being associated with chronic hyperglycemia which causes activation of multiple biochemical pathways which induces oxidative stress in diabetic neurons and leads to nerve damage and neuronal ischemia [44] and it can also be explained by possible late diagnosis.

In agreement with other reports, our data showed that physical inactivity was found to be an independent predictor of DPN [24, 45, 46]. The possible reason for this association might be that physical exercise can increase microvascular circulation, for release of neurotropic factors, attenuation of oxidative stress, and for physiological well-being of the body [47].

Finally, the present study found a significant association between smoking habit and DPN. Other studies have also found a similar association [32, 43]. The association between smoking and DPN can be explained by the fact that smoking causes neuropathy via neuronal ischemia from endothelial damage, increased inflammation, oxidative stress, interference with glucose metabolism, and from direct toxic effects on the neurons [48].

## 5. Limitation of the Study

Inferring casual association is difficult due to the cross-sectional nature of the study and lack of nerve conduction testing, which is the gold standard diagnostic test for confirmation of DPN diagnosis. The duration of diabetes as measured in this study might not reflect the true duration of the disease, but the time since diagnosis and actual diabetes onset might precede diagnosis in type 2 DM. The patient-recall bias may be affecting the performance of the MNSI symptom score, and the effect of glycemic control (HbA1C) was not studied. Finally, interrater and intrarater variability/variation during the examination version of MNSI was not calculated.

## 6. Conclusion

In our study, there was a high prevalence of DPN and physical inactivity, smoking, age above 40 years, and DM duration of above 5 years were the risk factors associated with it. Early detection and appropriate interventions are important for patients with age above 40 years, physically inactive, smokers, and those with DM duration of above 5 years.

## Figures and Tables

**Table 1:** Sociodemographic characteristics of patients with diabetes mellitus at JUMC 2019, Jimma, Ethiopia.

Variables	Category	Number	Percentage
Sex	Male	203	55.5
	Female	163	44.5

Age	<30 years	43	11.7
	30 to 39 years	30	8.2
	40 to 49years	87	23.8
	≥50 years	206	56.3

Marital status	Married	289	79
	Single	60	16.4
	Others^∗^	17	4.6

Religion	Muslim	162	44.3
	Orthodox	145	39.6
	Protestants	46	12.6
	Others^†^	13	3.6

Educational status	Illiterate	108	29.5
	Primary	167	45.6
	Secondary	44	12
	College and above	47	12.8

Occupational status	House wife	108	29.5
	Farmer	113	30.9
	Employer	78	21.3
	Private worker	51	13.9
	Others^‡^	16	4.4

Residence	Urban	177	48.4
	Rural	189	51.6

Family history of DM	Yes	87	23.8
	No	279	76.2

Monthly income (USD)	<29.5	96	26.2
	29.5 to 58.9	40	10.9
	58.93 to 88.4	95	26
	≥88.4	135	36.9

∗Widowed, divorced; ^†^catholic, wakefata; ^‡^retired, unemployed.

**Table 2:** Clinical and behavioral characteristics of patients with diabetes mellitus at JUMC 2019, Jimma, Ethiopia.

Variables	Category	Number	Percentage
Duration of DM	<5years	54	14.8
	5 to 10 years	68	18.6
	≥10 years	244	66.7

Treatment regimen	Insulin	23	6
	Noninsulin	284	77.6
	Both	23	6

BMI (kg/m^2^)	<18.5	30	8.2
	18.5 to 24.9	231	63.1
	25-29.9	84	23
	≥30	21	5.7

Alcohol intake	Current	44	12
	Former	31	8.5
	Never	291	79.5

Smoking	Current	54	14.8
	Former	68	18.6
	Never	244	66.7

Physical exercise	Physically active	179	48.9
	Physically inactive	187	51.1

FBS (mg/dl)	<200	263	71.9
	≥200	103	28.1

**Table 3:** Independent predictors of DPN among diabetic patients at JUMC 2019, Jimma, Ethiopia.

Variables	Category	DPN		Bivariate analysis		Multivariate analysis	
		Yes	No	*p* value	COR (95% CI)	*p* value	AOR (95% CI)
Age (years)	<30	7	36	1	1	1	
	30-39	1	29	0.115	0.177 (0.02, 1.525)	0.211	0.23 (0.025, 2.26)
	40-49	43	44	0.001	5.02 (2.02,12.5)	0.007^∗^	4.57 (1.50, 13.9)
	≥50	145	61	≤0.001	12.2 (5.15,28.9)	0.001^∗^	6.5 (2.24, 18.79)

Educational level	Illiterate	63	45	0.072	1.89 (0.945, 3.78)	0.787	0.88 (0.36, 2.16)
	Primary	89	78	0.195	1.54 (0.80, 2.96)	0.887	1.06 (0.467, 2.41)
	Secondary	24	20	0.254	1.62 (0.707, 3.71)	0.14	2.22 (0.754, 6.56)
	College and above	20	27	47	1	1	1

Marital status	Married	156	133	1	1	1	1
	Single	35	25	0.538	1.19 (0.68, 2.09)	0.444	0.73 (0.334, 1.61)
	Others†	5	12	0.058	0.355 (0.122, 1.03)	0.817	0.83 (0.17, 4.04)

Smoking	Current	45	9	≤0.001	6.96 (3.2, 14.87)	≤0.001^∗^	7.96 (3.23, 19.6)
	Former	49	19	≤0.001	3.59 (1.99, 6.46)	0.013^∗^	2.65 (1.22, 5.77)
	Never	102	142	1	1	1	1

Physical exercise	Physically active	75	104	1	1	1	1
	Physically inactive	121	66	≤0.001	2.54 (1.66, 3.87)	0.015^∗^	2.02 (1.14, 3.55)

Duration of DM(years)	<5	74	127	1	1	1	1
	5-10	55	35	≤0.001	2.69 (1.61, 4.49)	≤0.001^∗^	3.06 (1.63, 5.77)
	≥10	67	8	≤0.001	14.3 (6.54, 31.5)	≤0.001^∗^	7.19 (2.99, 17.2)

∗Value statistically significant; ^†^widowed, divorced; AOR: adjusted odds ratio; COR-Crude odds ratio; 1: reference.

## Data Availability

The data used to support the findings of this study are available from the corresponding author upon reasonable request.

## Abbreviations

AOR: Adjusted odds ratio
BMI: Body mass index
CI: Confidence interval
COR: Crude odds ratio
DM: Diabetes mellitus
DPN: Diabetic peripheral neuropathy
HIV: Human immune virus
IDF: International diabetes federation
JUMC: Jimma University Medical Center
MNSI: Michigan neuropathy screening instrument
WHO: World Health Organization

## References

[1] Amos A. F., McCarty D. J., Zimmet P. (1997). The rising global burden of diabetes and its complications: estimates and projections to the year 2010. *Diabetic medicine*.

[2] CfDCaPNDS Report (2014). *Estimates of Diabetes and Its Burden in the United States*.

[3] Feleke Y., Enquselassie F. (2005). An assessment of the health care system for diabetes in Addis Ababa. *Ethiopian Journal of Health Development*.

[4] Lange-maia B. S., Lange-maia B. S., Khatib O., Tabatabaei Malazy O. (2007). Prevention and public approach to diabetic foot. *Iranian J of Diabetes & Lipid Disorders*.

[5] Gebre M. W. (2013). Diabetes mellitus and associated diseases from Ethiopian perspective: systematic review. *Ethiopian Journal of Health Development.*.

[6] Candrilli S. D., Davis K. L., Kan H. J., Lucero M. A., Rousculp M. D. (2007). Prevalence and the associated burden of illness of symptoms of diabetic peripheral neuropathy and diabetic retinopathy. *Journal of Diabetes and its Complications*.

[7] Alleman C. J. M., Westerhout K. Y., Hensen M. (2015). Humanistic and economic burden of painful diabetic peripheral neuropathy in Europe: a review of the literature. *Diabetes Research and Clinical Practice*.

[8] Tesfaye S., Boulton A. J. M., Dyck P. J. (2010). Diabetic neuropathies: update on definitions, diagnostic criteria, estimation of severity, and treatments. *Diabetes Care*.

[9] Neuropathies D. The nerve damage of diabetes [article online]. http://nih.gov/dm/pubs/neuropathy.

[10] Kengne A. P., Echouffo-Tcheugui J. B., Sobngwi E., Mbanya J. C. (2013). New insights on diabetes mellitus and obesity in Africa–part 1: prevalence, pathogenesis and comorbidities. *Heart*.

[11] Kengne A. P., Sobngwi E., Echouffo-Tcheugui J. B., Mbanya J. C. (2013). New insights on diabetes mellitus and obesity in Africa-part 2: prevention, screening and economic burden. *Heart*.

[12] Vinik A. I. (2008). Diabetic neuropathies. In Controversies in Treating Diabetes. *Humana Press.*.

[13] Albers J. W., Pop-Busui R. (2014). Diabetic neuropathy: mechanisms, emerging treatments, and subtypes. *Current neurology and neuroscience reports.*.

[14] Sheffield S. T. D. (2012). Advances in the epidemiology, pathogenesis and management of diabetic peripheral neuropathy. *Diabetes/Metabolism Research and Reviews*.

[15] Bruschi L. K. M., da Rocha D. A., Filho E. L. G. (2017). Diabetes mellitus and diabetic peripheral neuropathy. *Open Journal of Endocrine and Metabolic Diseases*.

[16] Calle-Pascual A. L., Redondo M. J., Ballesteros M. (1997). Nontraumatic lower extremity amputations in diabetic and non-diabetic subjects in Madrid, Spain. *Spain. Diabetes & metabolism.*.

[17] Federation I. D. (2017). *IDF Diabetes*.

[18] McGill M., Molyneaux L., Yue D. K. (2005). Which diabetic patients should receive podiatry care? An objective analysis. *Internal medicine journal*.

[19] Abdissa D., Adugna T., Gerema U., Dereje D. (2020). Prevalence of Diabetic Foot Ulcer and Associated Factors among Adult Diabetic Patients on Follow-Up Clinic at Jimma Medical Center, Southwest Ethiopia, 2019: An Institutional-Based Cross-Sectional Study. *Journal of Diabetes Research*.

[20] Ejigu A. (2000). <b>Brief communication</b>: Patterns of chronic complications of diabetic patients in Menelik II hospital, Ethiopia. *Ethiopian Journal of Health Development*.

[21] Tesfaye S. (2011). Recent advances in the management of diabetic distal symmetrical polyneuropathy. *Journal of Diabetes Investigation*.

[22] Boulton A. J., Vinik A. I., Arezzo J. C. (2005). Diabetic neuropathies: a statement by the American Diabetes Association. *Diabetes Care*.

[23] Tesfaye S., Chaturvedi N., Eaton S. E. M. (2005). Vascular risk factors and diabetic neuropathy. *New England Journal of Medicine*.

[24] Jember G., Melsew Y. A., Fisseha B., Sany K., Gelaw A. Y., Janakiraman B. (2017). Peripheral sensory neuropathy and associated factors among adult diabetes mellitus patients in Bahr Dar, Ethiopia. *Journal of Diabetes & Metabolic Disorders*.

[25] Kiani J., Moghimbeigi A., Azizkhani H., Kosarifard S. (2013). The prevalence and associated risk factors of peripheral diabetic neuropathy in Hamedan, Iran. *Iran. Arch Iran Med.*.

[26] Iunes D. H., Rocha C. B. J., Borges N. C. S., Marcon C. O., Pereira V. M., Carvalho L. C. (2014). Self-care associated with home exercises in patients with type 2 diabetes mellitus. *PLoS One*.

[27] Riley L., Guthold R., Cowan M. (2016). The World Health Organization STEPwise approach to noncommunicable disease risk-factor surveillance: methods, challenges, and opportunities. *American Journal of Public Health*.

[28] Moghtaderi A., Bakhshipour A., Rashidi H. (2006). Validation of Michigan neuropathy screening instrument for diabetic peripheral neuropathy. *Clinical Neurology and Neurosurgery*.

[29] Herman W. H., Pop-Busui R., Braffett B. H. (2012). Use of the Michigan neuropathy screening instrument as a measure of distal symmetrical peripheral neuropathy in type 1 diabetes: results from the Diabetes Control and Complications Trial/Epidemiology of Diabetes Interventions and Complications. *Diabetic Medicine*.

[30] Al Washali A. Y., Azuhairi A. A., Hejar A. R., Amani Y. W. (2014). Prevalence and associated risk factors of diabetic peripheral neuropathy among diabetic patients in national center of diabetes in Yemen. *International Journal of Public Health and Clinical Sciences.*.

[31] Pop-Busui R., Lu J., Lopes N., Jones T. L., BARI 2D Investigators (2009). Prevalence of diabetic peripheral neuropathy and relation to glycemic control therapies at baseline in the BARI 2D cohort. *Journal of the Peripheral Nervous System*.

[32] Yeboah K., Puplampu P., Boima V., Antwi D. A., Gyan B., Amoah A. G. B. (2016). Peripheral sensory neuropathy in type 2 diabetes patients: A case control study in Accra, Ghana. *Journal of Clinical & Translational Endocrinology*.

[33] Mimi O., Teng C. L., Chia Y. C. (2003). The prevalence of diabetic peripheral neuropathy in an outpatient setting. *The Medical Journal of Malaysia*.

[34] Khawaja N., Abu-Shennar J., Saleh M., Dahbour S. S., Khader Y. S., Ajlouni K. M. (2018). The prevalence and risk factors of peripheral neuropathy among patients with type 2 diabetes mellitus; the case of Jordan. *Diabetology and Metabolic Syndrome*.

[35] Li L., Chen J., Wang J., Cai D. (2015). Prevalence and risk factors of diabetic peripheral neuropathy in type 2 diabetes mellitus patients with overweight/obese in Guangdong province, China. *Primary care diabetes*.

[36] Elbarsha A., Hamedh M. A., Elsaeiti M. (2019). Prevalence and risk factors of diabetic peripheral neuropathy in patients with type 2 diabetes mellitus. *Ibnosina J Med Biomed Sci*.

[37] Kisozi T., Mutebi E., Kisekka M. (2017). Prevalence, severity and factors associated with peripheral neuropathy among newly diagnosed diabetic patients attending mulago hospital: a cross-sectional study. *African Health Sciences*.

[38] Gill G., Gebrekidan A., English P., Wile D., Tesfaye S. (2008). Diabetic complications and glycaemic control in remote North Africa. *QJM*.

[39] Tilahun A. N., Waqtola C., Tewodros G. M., Amare D. W., Yohannis M. (2017). Major Micro vascular complications and associated risk factors among diabetic outpatients in Southwest Ethiopia. *Endocrinol Metab Syndr*.

[40] Janghorbani M., Rezvanian H., Kachooei A. (2006). Peripheral neuropathy in type 2 diabetes mellitus in Isfahan, Iran: prevalence and risk factors. *Acta neurologica scandinavica.*.

[41] Odoh G., Enamino M., Chuhwak E. K. (2017). Diabetic peripheral neuropathy and its risk factors : a community based study. *International Journal of Medicine & Biomedical Sciences*.

[42] Rota E., Quadri R., Fanti E. (2005). Electrophysiological findings of peripheral neuropathy in newly diagnosed type II diabetes mellitus. *Journal of the Peripheral Nervous System*.

[43] D’Souza M., Kulkarni V., Unnikrishnan Bhaskaran H. A. (2015). Diabetic peripheral neuropathy and its determinants among patients attending a tertiary health care centre in Mangalore. *India. Journal of public health research*.

[44] Edwards J. L., Vincent A., Cheng T., Feldman E. L. (2014). Diabetic neuropathy : mechanisms to management. *Pharmacology & Therapeutics*.

[45] Al Maskari F. (2014). Prevalence and determinants of peripheral neuropathy in patients with type 2 diabetes attending a tertiary care center in the United Arab Emirates. *Journal of Diabetes & Metabolism*.

[46] Sankari H., Vishnu R., Bazroy J., Singh Z. (2015). Prevalence and determinants of peripheral neuropathy among diabetics in a rural cum costal area of Villupuram district, Tamil Nadu. *International Journal of Research in Medical Sciences*.

[47] Kluding P. M., Pasnoor M., Singh R. (2012). The effect of exercise on neuropathic symptoms, nerve function, and cutaneous innervation in people with diabetic peripheral neuropathy. *Journal of Diabetes and its Complications*.

[48] Clair C., Cohen M. J., Eichler F., Selby K. J., Rigotti N. A. (2015). The effect of cigarette smoking on diabetic peripheral neuropathy: a systematic review and meta-analysis. *Journal of General Internal Medicine*.

